# The value of fever assessment in addition to the Early Detection Infection Scale (EDIS). A validation study in nursing home residents in Sweden

**DOI:** 10.1186/s12877-023-04266-6

**Published:** 2023-09-22

**Authors:** Pia Tingström, Nadine Karlsson, Ewa Grodzinsky, Märta Sund Levander

**Affiliations:** Medical Faculty, Linköping University, Linköping, Sweden

**Keywords:** Infection, Frail elderly, Fever, Assessment, EDIS

## Abstract

**Background:**

In order to improve detection of suspected infections in frail elderly there is an urgent need for development of decision support tools, that can be used in the daily work of all healthcare professionals for assessing non-specific and specific changes. The aim was to study non-specific signs and symptoms and fever temperature for early detection of ongoing infection in frail elderly, and how these correlates to provide the instrument, the Early Detection Infection Scale (EDIS), which is used to assess changes in health condition in frail elderly.

**Methods:**

This was an explorative, prospective cohort study, including 45 nursing home residents, 76 to 99 years, in Sweden. Nursing assistants measured morning ear body temperature twice a week and used the EDIS to assess individual health condition daily for six months. The outcome comprised events of suspected infection, compiled from nursing and medical patient records. Factor analysis and multivariate logistic regression analysis were performed to analyse data.

**Results:**

Fifteen residents were diagnosed with at least one infection during the six-month follow-up and 189 observations related to 72 events of suspected infection were recorded. The first factor analysis revealed that the components, change in cognitive and physical function, general signs and symptoms of illness, increased tenderness, change in eye expression and food intake and change in emotions explained 61% of the variance. The second factor analysis, adding temperature assessed as fever to > 1.0 °C from individual normal, resulted in change in physical function and food intake, confusion and signs and symptoms from respiratory and urinary tract, general signs and symptoms of illness and fever and increased tenderness, explaining 59% of the variance. In the first regression analysis, increased tenderness and change in eye expression and food intake, and in the second change in physical function and food intake, general signs and symptoms of illness and fever (> 1.0 °C from individual normal) and increased tenderness were significantly associated with increased risk for ongoing infection.

**Conclusion:**

No items in the EDIS should be removed at present, and assessment of fever as > 1.0 °C from individual normal is a valuable addition. The EDIS has the potential to make it easier for first line caregivers to systematically assess changes in health condition in fragile elderly people and helps observations to be communicated in a standardised way throughout the care process. The EDIS thus contributes to ensuring that the decisions not being taken at the wrong level of care.

## Background

The incidence of infections increases with age [1], especially in nursing home residents (NHRs). Due to general frailty and physical incapability [2–5], the elderly are at higher risk of morbidity and mortality, increased antibiotic usage and hospital admission [6–8]. Infection also causes long rehabilitation and functional decline [9, 10]. Signs and symptoms of infection in the frail elderly are often atypical. Specific ones, especially lacking increased temperature assessed as fever, are absent, causing delayed diagnosis, increased risk of under- or over-diagnosis [1, 11–13]. There is also an increased risk of over-treating with antibiotics [14, 15]. Infectious disease in NHRs requires a long recovery with the risk of persistent impaired physical function even after the infection has been cured [1, 7, 10].

Assessing the health status of frail elderly is difficult due to the complexity of ageing, frailty, and atypical presentation. To add to this challenge, elderly persons are unlikely to report symptoms to health-care providers, or to family members [10].

In addition, previous studies have concluded that early symptoms and signs of infection are very similar to symptoms and signs of acute illness other than infection [16, 17]. Boockvar et al. [18] reported that lethargy, weakness, and decreased appetite correctly predicted the presence of an acute illness in 50% of cases when the symptoms were reported. Agitation and disorientation or falls predicted an acute illness one in three times and one in four times, respectively.

Taken together, assessing the health status of frail elderly people is a challenge. In order to improve detection of suspected infections in frail elderly there is an urgent need for development of decision support tools, that can be used in the daily work of all healthcare professionals for assessing non-specific and specific changes [11, 19–28]. To develop a caregiver daily impression rating instrument for personal caregivers, Ae et al. [11] performed a retrospective review of care records and interviewed experienced long-term caregivers. They found body temperature ≥ 37.5 °C, “change in feeding,” “change in emotion,” “disengaged or listless gaze,” “decrease in eye reactivity” and “change in movement” as valid for assessing changes in the condition of frail elderly persons.

With the purpose of improving detection of suspected infections in frail elderly we developed the Early Detection of Infection Scale (EDIS). The items in the EDIS are based on what nursing assistants (NAs) think is a habitual condition and how it changes in case of suspicion of ongoing infection. NAs related suspected infection to non-specific signs and symptoms in two everyday phrases: “he/she is not as usual” and “he/she seems to be ill”. The first phrase described behavioural changes and discomfort, shown for example by eye expression, confusion, aggressiveness, infirmity/apathy, lack of restraint, restlessness, and changed food intake, while the second more distinctly related to well-established general signs and symptoms of respiratory or urinary tract symptoms, wounds or pain, fever, shaking, shivering, paleness, or flushed face [29]. Earlier results indicate that non-specific symptoms are strongly related to each other and, although not significantly, also to more specific symptoms of illness, i.e. “he/she seems to be ill”. However, none of the items in the EDIS appears to specifically verify the presence or absence of infection, i.e. they do not have sufficient sensitivity or specificity or solely correlate with infection. The item “general signs and symptoms of illness”, strongly correlated with several other items, such as “confusion”, “aggressiveness”, “infirmity/apathy”, “unrestrained behaviour”, “restlessness”, “urinary tract symptoms”, “respiratory tract symptoms” and “body temperature” [30]. Instead of a fixed threshold for temperature in fever, the item “increased body temperature” is based on that normal body temperatures in frail elderly show large variations, 33.8 to 38.4 °C for ear temperature and 35.6 °C to 38,0 °C for rectal temperature [31]. The same may be expected for increased temperatures in fever [32], Hence, fixed thresholds for fever is not accurate to use for assessing the individual [33]. Therefore, Sund Levander and Grodzinsky [34, 35] recently suggested defining temperature in fever as > 1 °C increase from individual normal body temperature.

In summary, our results are promising but items in the EDIS need to be further explored to exclude those that have no value for early detection of suspected infection. In addition, the value of fever as an item in EDIS is interesting to study.

Based on our former development of the EDIS instrument we aimed to detect (suspicion) of infections in frail elderly by.

1. Testing the current EDIS in a new group of frail elderly in a longitudinal cohort exploratory design.
2. Exploring if the EDIS could be modified with use of factor analysis and multiple regression analysis.
3. Explore if the EDIS could be improved by this new definition of fever.

## Methods

The study was an explorative, prospective cohort study.

### Sample

Nursing home residents from one municipal non-profit long-term facility with 56 beds in the South of Sweden were invited to participate According to earlier research of verified infections in a similar sample [36]we calculated an incidence of 28 verified infections in the available nursing home with 56 residents.

### Measurements

Background data about chronic disease and medication were collected from medical and municipal patient records by a research nurse. Physical status was assessed through interviews with the resident or NAs, using the Katz personal (P) and instrumental (I) ADL-indexes. The P-ADL has six categories: bathing, dressing, toileting, transfer, continence, and feeding, and the I-ADL has the categories of cooking, transportation, shopping, and cleaning. The residents were graded from 0 to 10, where 0 = independency in all variables and 10 = dependency in all variables. Grade 5 means dependency in all I-ADL and in one P-ADL activity [37, 38].

Before inclusion the research nurse measured morning body temperature in habitual condition, before getting up, and before medication, food and drink. Individual body temperature was established as the mean value of two morning ear temperatures. Temperature was measured in both ears with Genius 2 (Medtronic, Boston, MA, US).

To assess temperature as fever, we used both an increase of 1 °C above individual normal temperature [34, 39], and the traditional threshold for fever, i.e. 38 °C.

The EDIS has earlier been tested in a one-year follow-up when NAs used the scale to document episodes of suspected early signs and symptoms of infection in 204 NHRs [30]. Documented episodes were classified as “no infection”, “possible infection”, and “infection”. Content validity analysis showed that all the EDIS items, except pain, correlated significantly with at least one other statement. The construct validity showed that the items “increased temperature”, “respiratory symptoms” and “general signs and symptoms of illness” were significantly related to “infection” in 61%. Although the EDIS instrument did not have precision in predicting “possible infection”, it correctly predicted patients with “no infection” and “infection” in 67% and 61% of cases, respectively. Sensitivity and specificity were calculated for two of the items that it was possible to dichotomise, “respiratory symptoms” (51% and 29% respectively) and “general signs and symptoms of illness” (41% and 30% respectively).

Morning body temperature was also measured when required in conjunction with suspected infection, throughout the six-month follow-up period. All thermometers, provided by the research team for the study were calibrated and set to measure the actual temperature without predetermined additions for adjustment to another measurement site. Temperature assessed as fever was defined as at least 1^0^ C increase from individual normal body temperature.

### Outcome

Outcome was infection, verified with examination by the GP and/or including CRP documented in medical epicrisis. EDIS observations over several consecutive days were grouped together in events of suspected infections EDIS documentation There were 72 events, with 15 diagnosed as infection and 57 as no infection.

### Data collection

After inclusion, trained NAs used a paper version of EDIS to assess individual health due to condition daily and individual follow-up for six months. Data collection was performed from October 2017 to June 2018. If the individual was assessed to be “as usual”, i.e. in his/her habitual state, no further action was taken. If the NA observed changed behaviour, according to the items in the EDIS, and assessed the residents as “not as usual” or “seems to be ill”, compared to his/her habitual state, the body temperature in both ears was measured and the registered nurse (RN) was informed. The RN in turn acted by either waiting and following the course, initiating body temperature measurement, measuring CRP and urine sampling, or contacting a general practitioner (GP). Events that were related to suspected infection, as well as mortality were noted in the municipal health care patient record and epicrisis in connection with medical assessment and hospital care. A research nurse was available day time during the whole study to facilitate and oversee data collection. EDIS. The research nurse performed all data collection and registered data in the data base.

### Statistical analysis

Background variables are presented as proportions, m ± SD and range. Data investigating individual health status consists of a varying number of observations of early non-specific signs and symptoms per individual. The measurements include repeated observations for several separate events of suspected infections for everyone. A factor analysis was performed to derive simplified dimensions of health from the set of variables measuring early non-specific signs and symptoms of infection (EDIS) and temperature assessed as fever. Fever was defined as an increase of > 1.0 °C from the individual’s normal body temperature. Temperature data from the right ear was used as a larger variation in the left ear is reported [36]. The primary EDIS variables included in the analyses were “eye expression, unrestrained, aggressiveness, anxiety, confusion, infirmity/apathy, food intake, pain, general signs, respiratory symptoms, urinary tract symptoms, wound”. The extraction method was principal component analysis, and an orthogonal rotation was performed with Varimax and Kaiser normalisation using the option of replacement of missing values with the mean [40]. Two separate factor analyses were performed: (i) the first factor analysis included only the set of variables measuring early non-specific signs and symptoms of infection (EDIS); (ii) the second factor analysis included additionally the dichotomous variable fever, i.e. increase of > 1.0 °C from individual normal. Five composite dimensions were derived from each factor analysis. For each dimension of health, the factor scores were aggregated per event and subsequently used as independent variables in regression analyses.

A logistic regression with robust estimates of standard errors was then performed using infection as the primary outcome. The clustering effects of events within each participant were considered by calculating robust estimates of standard errors. A multivariate logistic regression analysis was performed to examine the independent effects of temperature assessed as fever and the five derived EDIS components from the first factor analysis. Then a second multivariate logistic regression was performed to examine the independent effect of the five others derived EDIS components including fever, i.e. an increase of > 1.0 °C from individual normal body temperature. The multivariate adjusted odds ratios with 95% confidence intervals were estimated in the logistic regression analysis. A level of 5% was statistically significant. Statistical analyses were performed in SPSS 28 and Stata 17.

## Results

The sample consisted of 45 NHRs, 76 to 99 years, 12 men and 33 women. All residents needed daily care and support, of whom 30% managed personal activities of daily living (ADL) with minor assistance. Backgrounds variables are presented in Table 1.

**Table 1:** Frequency of background factors in 45 elderly nursing home residents

Variable	m +/- SD
Age (years)	88 ± 6
ADL^a^	4 ± 4
Smoking (**n (%)**	4/9
**Chronic disease**	**n (%)**
Dementia	14 (31)
Heart disease	31 (69)
Asthma	5 (11)
Chronic obstructive pulmonary disease	3 (7)
Stroke	7 (16)
Diabetes	10 (22)
Chronic liver disease	2 (4)
Chronic kidney disease	2 (4)
Cancer	8 (18)
Autoimmune disease	1 (2)
Hypothyroidism	1 (2)
Hyperthyroidism	1 (2)
**Medication**	
Corticosteroids 5 mg or more daily	1 (2)
Sedatives	12 (27)
Antidepressants	16 (36)
Pain medication daily^b^	14 (31)
Anticoagulants	23 (51)

^a^ Activities of daily living. ^b^ Medication lowering temperature when fever, i.e. paracetamol and NSAID

Of the included 45 residents, 31 experienced 72 events of suspected infection. Fifteen residents (33%) were diagnosed with at least one infection during the six-month follow-up (range one to eight infections). In total, 189 observations related to these 72 events of suspected infection were recorded over time. Each event was a series of several observations during a short period (approximately one to two weeks). Of the total 189 observations, 165 were noted in EDIS and 24 were documented only in the RN files. Frequencies for each item in EDIS are presented in Table 2. There were 49 observations with an increase of > 1.0 °C from individual normal body temperature. If the usual fever definition (≥ 38 °C) was applied, 16 out of 49 (33%) measurements reached that level.

**Table 2:** Frequencies of Early Detection of Infection (EDIS) scale observations when infection was suspected in 45 nursing home residents

Item	All observations ^a^ n = 189	
**“He/she is not as usual”**	n	%
**Expression** (of illness) **in the eyes** (vacant/ hazy/ glassy/ roaming eyes)	10	5.3
**Unrestrained** (uncontrolled talk, in high spirits)	4	2.1
**Aggressiveness** (in talking and in actions)	3	1.6
**Anxiety** (over-excited, messy, does not sleep)	24	12.7
**Confusion** (muddled, increased signs of dementia, hallucination)	25	13.2
**Infirmity/Apathy** (drowsy, decreased mobility, needs more help)	81	42.9
**Food intake** (does not open mouth, less appetite, does not want to eat or drink)	29	15.3
**“He/she seems to be ill”**		
**General signs and symptoms of illness** (fever, hot/cold, shaking, shivering, pale, flushed face)	32	16 − 9
**Pain** **(**tenderness, moaning, tense body)	19	10.1
**Respiratory symptoms** (out of breath, cough, wheezing)	71	37.6
**Urinary tract symptoms** (often goes to toilet, stinging smell/ thick urine)	23	12.2
**Wound** **(**local redness and swelling, pus)	3	1.6

^a^A series of several observations during a short period grouped as one event

Two factor analyses were performed. The first one with the original 12 EDIS items and the second one with the addition of fever as an increase of > 1.0 °C from individual normal body temperature.

The first factor analysis identified five components with a factor loading of ≥ 0.5. All 12 EDIS items were included, two or three in each component. No item loaded with a factor loading of ≥ 0.5 in more than one component. The five components explained 61% of the variance. Items loading onto each component are presented in Table 3. The second factor analysis, adding difference of > 1.0 °C from individual normal body temperature as the 13^th a^ item, also resulted in five components that together explained 59% of the variance. In this factor analysis there were two items that did not load with a factor loading of ≥ 0.5 (“expressions of illness in the eyes” and “anxiety”). The other 11 items loaded otherwise in five factors compared to the first model. The five components with items are presented in Table 4.

**Table 3:** Factor analysis of items in Early Detection of Infection (EDIS) scale explaining the variance in signs and symptoms of infection in nursing home residents^†^

Items in EDIS. Observations of change in habitual condition	Components				
	Change in cognitive and physical function	General signs and symptoms of illness	Increased tenderness	Change in eye expression and food intake	Change in emotions
**Expression** (of illness) **in the eyes** (vacant/ hazy/ glassy/ roaming eyes)	0.108	0.095	0.073	**0.738**	− 0.121
**Unrestrained** (uncontrolled talk, in high spirits)	0.023	− 0.114	− 0.072	0.014	**0.772**
**Aggressiveness** (in talking and in actions)	0.230	0.029	0.023	− 0.044	**0.709**
**Anxiety** (over-excited, messy, does not sleep)	**0.731**	− 0.071	− 0.026	− 0.019	0.146
**Confusion** (muddled, increased signs of dementia, hallucination)	**0.706**	− 0.234	− 0.143	− 0.020	0.122
**Infirmity/Apathy** (drowsy, decreased mobility, needs more help)	**− 0.586**	− 0.325	− 0.245	0.437	0.080
**Food intake** (does not open mouth, less appetite, does not want to eat or drink)	− 0.284	0.013	0.012	**0.717**	0.117
**Pain (**tenderness, moaning, tense body)	− 0.047	− 0.146	**0.811**	0.196	− 0.042
**General signs and symptoms of illness** (fever, hot or cold, shaking, shivering, pale, flushed face)	0.043	**0.572**	− 0.148	0.311	− 0.128
**Respiratory symptoms** (out of breath, cough, wheezing)	− 0.106	**0.814**	− 0.135	− 0.110	− 0.125
**Urinary tract symptoms** (often goes to toilet, stinging smell/thick urine)	0.275	**− 0.581**	− 0.137	− 0.035	− 0.365
**Wound (**local redness and swelling, pus)	− 0.034	− 0.013	**0.804**	− 0.117	0.002

Number of observations = 189. Bold values are the items with high loading (> 0.50) characterising the component. †Carried out with Varimax rotation and eigenvalue threshold of > 1. Extraction Method: Principal Component Analysis. Rotation Method: Varimax with Kaiser Normalisation. Rotation converged in nine iterations

**Table 4:** Factor analysis of items in Early Detection of Infection (EDIS) scale with the addition of defining fever as ≥ 1 °C increase from individual normal body temperature explaining the variance in signs and symptoms of infection in nursing home residents

Items in EDIS. Observations ^a^ of change in habitual condition	Components				
	Change in physical function and food intake	Confusion and signs and symptoms from respiratory and urinary tract	General signs and symptoms of illness and fever^a^	Increased tenderness	Change in emotions
**Expression** (of illness) **in the eyes** (vacant/ hazy/ glassy/ roaming eyes)	0.479	0.026	0.162	0.094	− 0.039
**Unrestrained** (uncontrolled talk, in high spirits)	0.101	0.028	− 0.187	− 0.081	**0.747**
**Aggressiveness** (in talking and in doing)	− 0.091	0.088	0.050	0.021	**0.736**
**Anxiety** (over-excited, messy, does not sleep)	− 0.346	0.497	0.092	− 0.019	0.286
**Confusion** (muddled, increased signs of dementia, hallucination)	− 0.267	**0.652**	0.122	− 0.106	0.237
**Infirmity/Apathy** (drowsy, decreased mobility, needs more help)	**0.774**	− 0.046	− 0.113	− 0.183	− 0.053
**Food intake** (does not open mouth, less appetite, does not want to eat or drink)	**0.723**	− 0.124	0.098	0.061	0.095
**Pain (**tenderness, moaning, tense body)	0.156	0.038	− 0.076	**0.826**	− 0.043
**General signs and symptoms of illness** (fever, hot or cold, shaking, shivering, pale, flushed face)	0.107	− 0.175	**0.766**	− 0.071	− 0.067
**Respiratory symptoms** (out of breath, cough, wheezing)	− 0.289	**− 0.698**	0.258	− 0.212	− 0.074
**Urinary tract symptoms** (often goes to toilet, stinging smell/thick urine)	− 0.016	**0.628**	− 0.204	− 0.098	− 0.354
**Wound (**local redness and swelling, pus)	− 0.129	− 0.061	− 0.050	**0.804**	− 0.008
**Temperature** (> 1 degree C increase from normal body temperature)	0.050	0.039	**0.804**	− 0.059	− 0.020

^a^ Number of observations = 189. Bold values are the items with high loading (> 0.50) characterising the component. †Carried out with Varimax rotation and eigenvalue threshold of > 1. Extraction Method: Principal Component Analysis. Rotation Method: Varimax with Kaiser Normalisation. Rotation converged in seven iterations

Finally, two multivariate logistic regression analyses were performed to examine the independent effect of the five components found in each factor analysis as determinants for infection. The first regression analysis (Table 5) with the five components presented in Table 3 was statistically significant as a model (P = 0.032). Two of the components in the model were statistically significantly associated with increased risk for ongoing infection: “increased tenderness” (P = 0.008) and “change in eye expression and food intake” (P = 0.008). The overall p-value for the second model (Table 5) with the five components presented in Table 4 was not statistically significant (P = 0.109). However, three of the components in the second model were statistically significantly associated with ongoing infection: “change in physical function and food intake” (P = 0.022), “general signs and symptoms of illness and fever” (0.018) and “increased tenderness” with (P > 0.012).

The overall p-value of the logistic regression model presented in Table 5 was not statistically significant (P = 0.109). Since dependent variables in the model were estimated with factor analysis, the hypothesis that multicollinearity occurs can be excluded. Therefore, a sensitivity analysis was performed by reanalysing the second model including only the dependent variables that were statistically significant in Table 5 to increase the statistical power of the model. The dependent variables included were component “change in physical function and food intake”, component “general signs and symptoms of illness and fever”, and component “increased tenderness”. In the new analysis, odds ratios remained similar with data presented in Table 5: component “change in physical function and food intake” OR = 2.40 95% CI 1.08–5.34; component “general signs and symptoms of illness and fever” OR = 2.31; 95% CI 1.10‒4.87; and component “increased tenderness” OR = 1.67 95% CI = 1.10–2.54. The overall p-value of the parsimonious model became almost statistically significant (p = 0.052). This suggests that the model presented in Table 6 was not statistically significant due to the low statistical power related to the small number of observations.

**Table 5:** Logistic regression of components and included items related to increased risk of infection in nursing home residents. Number of observations = 72. Prob > chi2 **=** 0.032

Components	Items in EDIS	Odds ratio	P value	95% CI^a^	
Change in cognitive and physical function	Anxiety, confusion, infirmity/apathy	1.26	0.434	0.71	2.23
General signs and symptoms of illness	General signs and symptoms of illness Respiratory symptoms Urinary tract symptoms	1.14	0.709	0.58	2.24
Increased tenderness	Pain, wound	1.68	**0.008**	1.15	2.47
Change in eye expression and food intake	Eye expression, food intake	4.47	**0.008**	1.47	13.60
Change in emotions	Unrestrained aggressiveness	0.82	0.562	0.41	1.62

^a^ CI = Confidence interval

**Table 6:** Logistic regression of components, included items and fever^b^ related to increased risk of infection in nursing home residents. Number of observations = 72. Prob > chi2 0.109

Components	Items in EDIS	Odds ratio	P value	95% CI^a^	
Change in physical function and food intake	Infirmity/ apathy food intake	2.66	**0.022**	1.15	6.15
Confusion and signs and symptoms from respiratory and urinary tract	Confusion Respiratory symptoms Urinary tract symptoms	1.49	0.350	0.65	3.43
General signs and symptoms of illness and fever^b^	General signs and symptoms, fever^b^	2.43	**0.018**	1.17	5.06
Increased tenderness	Pain, wound	1.76	**0.012**	1.13	2.75
Change in emotions	Unrestrained Aggressiveness	0.91	0.758	0.48	1.70

^a^CI=Confidence interval ^b^ > 1 degree C above normal

## Discussion

Since the last testing of the EDIS instrument [30] we have further explored it with factor analysis and multiple regression analysis in a new group of frail elderly. We argue that no items in EDIS should be removed at present, and that assessment of fever as an increase of > 1.0 °C from individual normal body temperature is a valuable addition.

The first model in the present results (Table 5) was based on the original version of EDIS and resulted in five useful components, which we have given new names. In the second model (Table 6) we expanded the instrument by including an extra item; a new definition of fever temperature, i.e. an increase of > 1.0 °C from individual normal body temperature. We found that both variants of EDIS are valuable in clinical practice as both are associated with increased risk for ongoing infection. Even though we have several observations with repeated measurements over a long period, the results must be considered preliminary. This means that the instrument has the potential to detect infection but must still be tested on a larger group with more events of infection to achieve sufficient power in the analyses.

All items in EDIS are based on NAs’ experience and are expressed with the descriptions and languages they used to communicate changed health condition in NHRs [29, 30, 41]. We believe that using everyday, easy to understand language in EDIS stimulates accurate and standardised clinical assessment of the health status of frail elderly people. Some items seem to be more frequently used than others, but none can at this stage be excluded. They all contribute to both models.

The symptoms lethargy, weakness, decreased appetite, agitation, disorientation and falls have previously been described as predicting acute illness [18]. The components “change in physical function and food intake”, “change in eye expression and food intake” and “change in emotions”, are in line with Ae et al. [11], who reported the components “change in feeding,” “change in emotion, ”disengaged or listless gaze” “decrease in eye reactivity” and “change in movement” as reflecting daily caregivers’ impression of illness latency. The component “change in eye expression” did not fit in our second model, perhaps as a the result of adding fever as an increase of > 1.0 °C from individual normal body temperature. Ae, [11] also reported that the observation “decrease in eye reactivity” was associated with eventual hospitalisation, which suggests that the item should not be excluded at this stage of developing the EDIS instrument.

A common observation when frail elderly suffer from infection is that fever is absent, which delays diagnosis and treatment [1, 11–13]. Because normal body temperature shows large variations, it is reasonable that the same should hold true for increased temperature in fever [32, 34, 35]. When the traditional cut-off for fever, i.e. ≥ 38 °C, was applied, only 33% were assessed as febrile. This indicates that assessing fever as an increase of > 1.0 °C from individual normal body temperature would improve detection of ongoing infection early on.

In previous studies we used one morning temperature to define baseline body temperature [31, 33, 34]. However, we found that body temperature varies when measured the same time for several days [36] we decided to use the mean value of two measurements of morning ear temperature to define individual normal.

In the present study we also intended to measure CRP directly at the nursing home when infection was suspected, but due to too few measurements (n = 11), we omitted this result in our analyses. Liu [42] reported that a CRP cut-off value of 60 mg/l had the best combination of sensitivity (80.7%,) and specificity (96.0%,), a positive predictive value of 91.9% and a negative predictive value of 89.8% for diagnosing bacterial infection in frail elderly. Hence, CRP seems to be a convenient and useful biomarker to detect bacterial infection in older patients, especially when other markers are atypical or not present [1, 42]. More studies are needed to verify whether lower cut-offs for CRP better predict ongoing infection in fragile elderly.

The purpose of the EDIS as a decision support tool is not to remedy/treat medically but to facilitate the step before diagnosis, so it can take place more quickly. We have previously reported that NAs in elderly care did not report their observations further or gave up, as they did not expect RNs to act further [41]. Hence, it seems random whether the changed condition is communicated further from NAs to RNs / GPs or not, depending on caregivers’ personal experience and performance. We also found that NAs also said that they sometimes added a few tenths to the temperature to get a response from the RN [41]. The consequence is that decisions on necessary measures remain at the wrong level of care.

## Conclusion

In conclusion, the results from the present pilot study suggests that no items in EDIS should be removed at present, and assessment of fever as an increase of at least 1.0 °C from individual normal is a valuable addition. The EDIS has the potential to make it easier for NAs, i.e. first line caregivers, to systematically assess changes in the health condition of fragile elderly people. The EDIS also support that the observation is communicated in a standardised way further in the care process, i.e. to RNs and then to GPs. The EDIS thus contributes to decisions not being taken at the wrong level of care.

### Limitations

We gathered data from one nursing home and included 45 out of 56 residents. In the follow-up time of six months there were few documented infections. This might be due to a relatively good physical and cognitive ability in the included sample. We could only include those who gave informed consent, and this should be considered when generalizing to frail elderly in other nursing homes. The fact that the overall p-value for the second regression (Table 5) was not statistically significant might be due to low statistical power due to the small number of infections. Interestingly, though, in the parsimonious version of each model (using as few variables as needed), the overall p-value decreased. In the second model (Table 6) the p-value in Table 5 decreased from 0.109 to 0.052.

Further research is needed to clinically investigate the possibility of the EDIS to detect suspected infection early, and hence decrease the time to diagnosis and treatment. A challenge is also how to implement this evidence-based new way to assess fever in clinical practice.

## Data Availability

The datasets used and/or analysed during the current study are available from the corresponding author on reasonable request.
